# Herpes Laryngis

**Published:** 1885-01

**Authors:** 


### Selections.

### Herpes Laryngis.

Dr. S. H. Chapman, in the *New York Medical Journal*, relates the histories of five cases of herpes laryngis, a disease of rare occurrence. These cases presented the following symptoms: Pain and soreness in the throat, cough, hoarseness, pain in swallowing, slight dyspnœa and great nervous agitation and anxiety. Physical examination showed the presence of herpetic ulcers in some part of the larynx, especially on the epiglottis, with an occasional one in the pharynx. The duration of the disease was from a few days to several weeks. In some cases there were several relapses. The treatment consisted of quinine, arsenic and strychnia, and the local use of inhalations of tincture of benzoin, oil of eucalyptus, oil of hops and the application of glycerine, glycerine with borax, and a weak solution of nitrate of silver. In some it was found necessary to administer anodynes.

From these five cases the author drew the following conclusions:

"1. There exists such a disease of the larynx as herpes.

"2. Its character is that of a neurosis.

"3. It is closely allied to herpes of the pharynx and other mucous membranes.

"4. It differs from other forms only on account of the peculiar microscopic anatomy of the larynx.

"5. It is peculiarly a disease of malarious districts, and one of the eccentric developments of malaria.

"6. It simulates tubercular inflammation of the epiglottis. The differential diagnosis, however, is easy. It is based upon the extreme rapidity of development, the absence of fever, the history of malarial affections, the previous or simultaneous development of herpetic eruption elsewhere and the rapid disappearance of the disease.

"7. Its seat is usually the posterior surface of the epiglottis.

"8. The nervous system is always profoundly affected."

